# Case Report: When access is limited: using CYP3A4 inhibition to maintain clinical benefit from elexacaftor/tezacaftor/ivacaftor

**DOI:** 10.3389/fphar.2026.1877957

**Published:** 2026-07-08

**Authors:** Keirran Hiscock, Michelle Wood, Ellie Johnson, Daniel Smith, Ieuan E. S. Evans

**Affiliations:** 1 Adult Cystic Fibrosis Centre, The Prince Charles Hospital, Brisbane, QLD, Australia; 2 Faculty of Medicine, University of Queensland, Brisbane, QLD, Australia

**Keywords:** CFTR modulator therapy, cystic fibrosis, elexacaftor/tezacaftor/ivacaftor (ETI), pharmacokinetic manipulation, therapeutic drug monitoring (TDM)

## Abstract

**Introduction:**

Cystic fibrosis transmembrane conductance regulator modulators (CFTRm), particularly elexacaftor/tezacaftor/ivacaftor (ETI), have transformed outcomes for people with cystic fibrosis (pwCF). However, access remains limited globally due to genotype-based eligibility and high medication cost. Pharmacokinetic manipulation using cytochrome P450 3A4 (CYP3A4) inhibitors has emerged as a potential strategy to extend ETI exposure in resource-constrained settings.

**Case:**

We describe a 52-year-old female with CF (G542X/T1246I) whose genotype initially precluded subsidised ETI access in Australia. She experienced progressive clinical decline in 2024, with worsening bronchiectasis, recurrent *Pseudomonas aeruginosa* exacerbations, and a fall in ppFEV1 below 40%. Supported by *in vitro* evidence of T1246I responsiveness, she self-funded ETI, resulting in rapid symptomatic improvement, reduced sputum burden, and objective gains in lung function, sweat chloride, and weight. To improve affordability, azithromycin was replaced with clarithromycin, a potent CYP3A4 inhibitor, enabling a reduced ETI dosing schedule. Therapeutic drug monitoring demonstrated lower serum concentrations of all ETI components compared with full-dose therapy, yet clinical stability and improved quality of life were maintained, with no further exacerbations. Clarithromycin was selected due to its established safety profile and dual utility as macrolide therapy, avoiding the hepatotoxicity risk associated with azole antifungals.

**Conclusion:**

This report supports the potential for CYP3A4 inhibition to aid with personalised ETI dosing strategies, particularly in low and middle-income countries where CFTRm access remains limited. Further research is needed to define optimal manipulation strategies, understand inter-individual variability in CFTRm metabolism, and evaluate the feasibility of therapeutic drug monitoring in diverse healthcare settings.

## Introduction

The management of people with CF (pwCF) has been transformed by the addition of cystic fibrosis transmembrane conductance regulator modulators (CFTRm). The most marked improvements in clinical, microbiological and functional capacity have been seen with triple therapy CFTRm including elexacaftor/tezacaftor/ivacaftor (ETI) (Trikafta®, Vertex Pharmaceuticals) and, more recently, vanzacaftor/tezacaftor/deutivacaftor (Alyftrek®, Vertex Pharmaceuticals). ETI specifically has been associated with marked improvements in lung function, reduction in exacerbation frequency, reduced sweat chloride levels and increased quality of life parameters (Middleton et al., 2019). However, access to ETI has been limited in part by genotype with eligibility requirements being for pwCF to have at least one F508del mutation when initially listed by the Pharmaceutical Benefits Scheme (PBS) in Australia in 2022. This access was subsequently widened to incorporate a larger number of genotypes in July 2025, with PBS criteria being modified to ‘at least one mutation in the *CFTR* gene that is considered responsive to elexacaftor/tezacaftor/ivacaftor potentiation based on clinical and/or *in vitro* assay.’

Globally, access to CFTRm medications such as ETI is heterogenous, with the high cost of patented medication being beyond the healthcare budget of many low and middle-income countries (LMIC). In response, innovative strategies to provide access to medications have been explored including the co-prescription of cytochrome P450 3A4 (CYP3A4) inhibitors to impair metabolism of CFTRm thereby extending medication half-life, reducing the therapeutic dose and consequently the cost of treatment (Zampoli et al., 2023).

Here we describe the experience of a pwCF without access to ETI at initial PBS listing in 2022 owing to her *CFTR* genotype G542X/T1246I. Following the expansion of PBS approval for ETI in 2025, our pwCF became eligible for subsidised access. However, prior to this she self-funded ETI for several months due to escalating symptom burden and clinical deterioration. Pharmacokinetic manipulation with clarithromycin, a potent CYP3A4 inhibitor, allowed for extended use of ETI at a reduced dose with maintained clinical benefit. This report supports the hypothesis of using CYP3A4 inhibition as a potential mechanism to reduce the cost of CFTRm therapy in resource restricted healthcare systems though wider evaluation would be required to determine inter-individual consistency.

## Case

We present the case of a 52-year-old female under the care of our tertiary CF centre who was diagnosed with CF at the age 43. *CFTR* genetic testing with whole exome sequencing reported a rare combination of mutations, G542X/T1246I, with an associated phenotype not further defined in the CFTR2 database. While T1246I is often considered a variant of varying consequence (CFTR2, 2026), the pwCF exhibited phenotypic characteristics consistent with CF including an indeterminate/borderline sweat chloride, moderate to severe upper lobe predominant bronchiectasis on computed tomography (CT), reduction in lung function (as measured by forced expired volume in second, FEV1), and chronic infection with *Pseudomonas aeruginosa* since at least 2014. She was pancreatic sufficient.

At the time of diagnosis in 2015 her genotype precluded the use of any available CFTRm therapy. She was additionally not eligible for ETI at the time of its initial PBS listing in 2022. Her health remained relatively stable following diagnosis until early 2024 when she started to show a progressive decline in spirometry, with FEV1% predicted (ppFEV1) dropping below 40% for the first time, which was attributed to increased mucous plugging and repeated infective pulmonary exacerbations. In response to deteriorating health and supported by *in vitro* evidence of responsiveness of T1246I to ETI (Bihler et al., 2024), a request for compassionate access to ETI was submitted. Of note, her G542X allele, being a class 1 mutation, was felt unlikely to achieve improved functionality with ETI therapy. In parallel, our pwCF explored self-funding ETI therapy and committed to purchasing an initial 1-month supply to determine whether treatment would result in symptomatic benefit alongside standard CF treatments, including a regimen of airway clearance supported by dornase alfa inhalation solution, hypertonic saline 6% nebulised solution, inhaled salbutamol and inhaled tobramycin (Tobi Podhaler®, Viatris). Monitoring of her response to ETI was conducted in-line with standards of clinical care and included clinical spirometry, weight and sweat chloride assessments. In addition, the pwCF consented to participate in a local pharmacokinetic (PK) research project with aims of developing a therapeutic drug monitoring (TDM) program for ETI (HREC/2024/MNHA/105142). TDM was undertaken at Pathology Queensland, Brisbane, Australia, using assays of ETI and its key constituent metabolites, developed and externally validated according to National Association of Testing Authorities (NATA) standards. The analytes of interest were assayed using an ultra-performance liquid chromatography tandem mass spectrometry method. In the concentration range from 0.01 to 20.0 mg/L, calibration curves were linear with a correlation coefficient >0.999 for all analytes.

**FIGURE 1:**
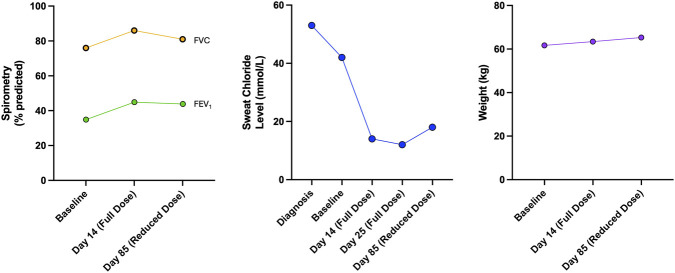
Clinical parameters following initiation of ETI from baseline, day 14, day 25 and day 85 of therapy. ppFEV1 improved from 35% to 45% at day 14 and was maintained at 44% at day 85. Weight increased from 61.7 kg to 63.4 kg at day 14 and 65.3 kg at day 85. Sweat chloride reduced from a baseline level of 42 mmol/L reaching a nadir of 12 mmol/L at day 25 and remained normal at 18 mmol/L at day 85.

Prior to commencement of ETI the pwCF was admitted to hospital and commenced on intravenous (IV) antibiotics (meropenem and tobramycin) for treatment of *P. aeruginosa*. On day 3 of IV antibiotics therapy ETI was commenced without complication. Within 2 weeks of ETI initiation, she noted a marked improvement in her quality of life and energy levels, reduced sputum volume and was subsequently able to return to a program of regular exercise. Objective measures demonstrated a reduction in sweat chloride, with increased FEV1 percentage predicted (ppFEV1) and weight (Figure 1).

**FIGURE 2:**
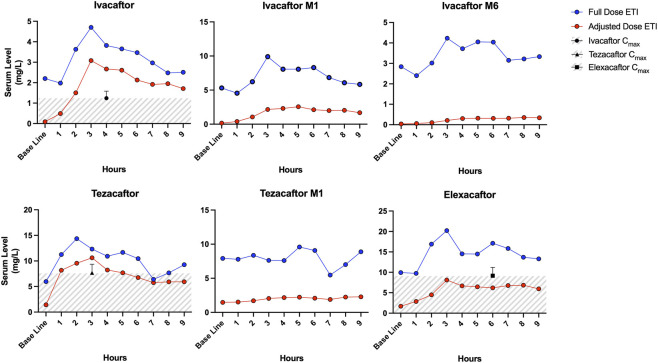
Therapeutic drug monitoring was conducted on both full dose (blue) and adjusted dose (red) ETI during our pwCF treatment course. Serum levels of elexacaftor, tezacaftor, ivacaftor and their associated major metabolites were analysed hourly for 9 hours following ingestion of ETI. Significant reductions in all constituent parts of ETI was noted. *C*_max_ levels were obtained from the Therapeutic Goods Administration product information for ETI to provide context and guidance on serum levels in the setting of no validated TDM program for ETI in Australia (Therapeutic Goods Administration, 2026).

Following completion of the first month of ETI therapy, financial considerations regarding ongoing self-funding of ETI became apparent. Consequently, switching prescribed macrolide therapy from azithromycin to clarithromycin was proposed to leverage CYP3A4 inhibition as a mechanism to slow ETI metabolism, reduce dosing frequency and lower direct treatment costs, consistent with previously published data from a South African cohort of pwCF (Zampoli et al., 2025). Azithromycin was therefore discontinued, and she was commenced on clarithromycin 250 mg BD alongside her ETI with close observation of her clinical status. This dose was selected based on the experience from the South African cohort (Zampoli et al., 2023). The addition of clarithromycin coincided with a reduction in ETI dosing as recommended by the ETI product information to two combination tablets (elexacaftor 100 mg/tezacaftor 50 mg/ivacaftor 75 mg) twice per week (Monday/Thursday). Repeat TDM was conducted after a month on this reduced dosing strategy as this was felt likely to provide adequate time for steady-state concentrations to be achieved. TDM was carried out to coincide with a day of ETI administration to allowing for optimal sampling standardisation to be achieved enabling a comparison between the two episodes of TDM to made. On both occasions TDM was conducted, ETI was administered after the pre-dose baseline blood sample had been collected (Figure 2).

A reduction in serum levels of all constituent parts of ETI, and their associated metabolites, was demonstrated compared to PK measurements completed on full-dose therapy. Area under the curve (AUC) for all constituent parts including metabolites showed reductions: ivacaftor full dose ETI 29.06, adjusted dose ETI 17.25; ivacaftor M1 full dose ETI 63.64, adjusted dose ETI 15.53; ivacaftor M6 full dose ETI 30.92, adjusted dose ETI 2.16; tezacaftor full dose ETI 92.65, adjusted dose ETI 66.32, tezacaftor M1 70.98, adjusted dose ETI 17.76; elexacaftor full dose ETI 134.1, adjusted dose ETI 52.21. Nevertheless, both clinical parameters and subjective wellbeing remained stable. The pwCF continued combination of ETI and clarithromycin for a total of 119 days until the PBS extended approval of ETI allowed a transition to PBS funded therapy owing to her genotype. Following transition to PBS funded ETI, clarithromycin was discontinued, and azithromycin was re-initiated 14 days later and ETI returned to full dose. To-date, in the 18 months since ETI initiation she has not required any further oral or IV antibiotic therapy and her most recent FEV1 was 54% predicted.

With the initiation of ETI, monitoring of her liver function tests (LFTs) was conducted on a monthly basis as per our local safety monitoring protocol. There was no change in bilirubin, alanine aminotransferase (ALT) and aspartate aminotransferase (AST) with all remain within the normal parameters throughout (data not shown).

## Discussion

Pharmacokinetic drug-to-drug interactions involving CYP-mediated metabolism are relatively common, given ETI is largely hepatically metabolised. Interactions with strong CYP3A4 inducers have been well described in the literature, particularly in the context of antibiotics such as rifabutin used to treat pathogens like *Mycobacterium abscessus* (Purkayastha et al., 2023; Mou et al., 2025; Sanders et al., 2026). However, the constituent parts of ETI are not equally influenced by CYP3A4 induction, with ivacaftor being shown to be particularly sensitive to CYP3A4 induced metabolism. Guidance on the use of strong CYP3A4 inducers in combination with ETI is that they should generally be avoided. Conversely, the guidance on the use of CYP3A4 inhibitors is less clearcut, though there is certainly evidence of increases in steady state serum levels of ivacaftor with the use of both ritonavir (a protease inhibitor), itraconazole (an antifungal) and clarithromycin (as utilised in our case) (Harrison et al., 2015; van der Meer et al., 2021; Liddy et al., 2017). Increases in serum levels of ETI could in theory predispose to increases in side-effect burden or intolerances, thereby necessitating ETI dose reductions. However, the degree to which this occurs has only been evaluated in limited circumstances (Zampoli et al., 2025). In our case, the initiation of full dose ETI correlated in improved clinical parameters including a reduction in sweat chloride, improved spirometry (FEV1) and increases in weight. Whilst some of these improvements may have in part been related to concurrent IV antibiotic therapy administration at initial commencement, the impact of sweat chloride in particular, was more in keeping with a direct impact of ETI. Additionally, on two sputum samples collected 2 and 4 weeks following ETI initiation she remained culture positive for *P. aeruginosa*. The later addition of clarithromycin, and subsequent dosing adjustment of ETI, saw a fall in all constituent parts of ETI. The metabolite elexacaftor-M23, not analysed by our assays, may also have been impacted in this scenario in a similar nature to other metabolites. However, this did not appear to be associated with a reduction in efficacy, with spirometry, sweat chloride levels and weight all remaining at a stable improved levels 2 months after initiation of pharmacokinetic manipulation via CYP3A4 inhibition. Importantly, she continued to report a marked improvement in self-reported quality of life, with complete resolution of her previously frequent exacerbations.

The choice of clarithromycin as the agent utilised to manipulate ETI dosing via CYP3A4 inhibition was guided primarily by its established safety profile and its utility as an alternative macrolide therapy to azithromycin. Antifungal azole therapy was initially considered. However, given no clinical indication, such as concurrent fungal infection, and the greater potential for drug-induced liver injury (DILI) with azole therapy, the risk was deemed to be too significant. This was especially the case given our local experience with higher rates of marked DILI within our CF cohort (Johnson et al., 2025). In contrast, macrolide therapy, in the form of azithromycin, has formed a cornerstone of CF care given its association with immunomodulation and anti-inflammatory effects (Wolter et al., 2002). Azithromycin is not a potent CYP3A4 inhibitor and, therefore, although the relative clinical efficacy of clarithromycin compared to azithromycin in CF remains uncertain, the advantage of sustaining ETI exposure was considered to outweigh any perceived risk related to reduced macrolide efficacy. Having been on a stable dose of azithromycin for several years it was felt that there was unlikely to be any intolerance in our pwCF with transitioning to clarithromycin when used in this setting. However, this would be a particularly important consideration to evaluate in a macrolide naïve pwCF with a theoretical increased risk of adverse drug reactions. Additionally, it is worth considering that there are wider implications of utilising macrolide antibiotics for the purposes of pharmacokinetic manipulation, particularly from an antimicrobial stewardship perspective and the risk of infections such as *Clostridium difficile*. Other potential risks relate to increased cardiovascular mortality (including QT prolongation and arrhythmia development, liver dysfunction, tinnitus and gastrointestinal upset (diarrhoea/nausea). However, the potential longer-term benefits in this case were felt to outweigh the risk of side-effect development particularly given the uneventful use of azithromycin for many years prior.

While our pwCF was fortunate to be able to access publicly funded and subsidised ETI following its extended approval via the PBS, this is not always the case globally. There remains significant health inequality in LMIC with access to CFTRm severely limited by their substantial cost. Approaches similar to those used in our case, and in the South African experience, may provide feasible interim strategies until more equitable access to CFTR modulators becomes a reality and should form the basis of ongoing evaluation around optimised implementation (Zampoli et al., 2025). It is worth considering that this isolated case has many limitations when conferring effects more widely, with inter-individual variability likely to be significant from a drug metabolism, drug-tolerance and individual dose adjustment perspectives. Additionally, it is important to recognise that the initiation of a CYP3A4 inhibitor to manipulate ETI in the manner conducted in our case is not standard practice in high-income countries. Furthermore, access to validated TDM assays to allow for dose monitoring and tailored adjustment are again unlikely to be universally accessible but may be able to help dosing strategies accordingly, the caveat being that TDM for CFTR modulator therapies is not currently part of routine clinical care, though interest in this area is growing (Jackson et al., 2026). The cost implications again are likely to be variable dependent on the healthcare system and reimbursement models in place. Other indirect cost reductions may also be apparent such as reduced pharmacy labour with reduced dosing, reductions in hospital admissions, and reductions in the use of IV antibiotic therapy. However, these may be offset somewhat by the requirement for more close monitoring in terms of hepatic and cardiovascular risk with clarithromycin usage and it remains the case that the high cost of CFTR modulator therapies mean that achieving cost neutrality, or indeed cost savings, may be challenging. Alternative strategies to reduce costs in LMIC include dose reductions without CYP3A4 manipulation, with evidence existing of maintained therapeutic benefit at reduced dose ETI (Spoletini et al., 2022; Hong et al., 2023). However, these dosing reduction strategies would still likely confer higher healthcare costs compared to CYP3A4 inhibition due to the dosing frequencies involved.

Finally, it is likely that numerous host-related factors will impact on the degree to which CFTRm are affected by CYP3A inducers or inhibitors, making management of such pwCF particularly challenging. These factors remain largely unstudied but are likely to include, but not be limited to, aspects such as absorption from the gut, renal excretion, the presence of liver disease, which is common in CF. Adjusting the dose of a CYP3A4 inhibitor upwards, within recommended dosing parameters, could be considered in pwCF receiving therapies with significant drug-drug interaction potential who exhibit a loss of therapeutic efficacy despite optimised adherence to treatment. Further understanding of these parameters is key to optimise the treatment of all pwCF, but particularly in LMIC settings where diverse strategies may be required to maximise clinical benefit under constrained economic conditions.

## Data Availability

The original contributions presented in the study are included in the article/supplementary material, further inquiries can be directed to the corresponding author.
